# Clinical Observation of Silicon Hydrogel Contact Lens Fitted Immediately after Small Incision Lenticule Extraction (SMILE)

**DOI:** 10.1155/2020/2604917

**Published:** 2020-08-19

**Authors:** Jifang Wang, Shuxin Xi, Bingjie Wang, Zhi Chen, Ke Zheng, Xingtao Zhou

**Affiliations:** ^1^Eye & ENT Hospital, Fudan University, Shanghai, China; ^2^Key Laboratory of Myopia, Ministry of Health, Fudan University, Shanghai, China

## Abstract

**Purpose:**

To examine the immediate use of bandage contact lenses (BCLs) for improving patient comfort after small incision lenticule extraction (SMILE) surgery.

**Methods:**

This is a prospective randomized controlled study in which one hundred and seventy-eight patients undergoing SMILE were randomly allocated to three groups: group A wore BCLs for 8 hours postsurgery, group B wore BCLs for 24 hours postsurgery, and group C did not wear any BCLs postsurgery. Eight subjective symptoms including photophobia, tearing, pain, foreign body sensation, burning, blurred vision, sting, and dry eyes were prospectively evaluated at 2 hours, 4 hours, 8 hours, and 24 hours, using a questionnaire with a total score of 24. The scores of symptoms and signs were compared between the three groups.

**Results:**

There was a statistically significant time effect on scoring, which implicated a decline in symptoms over time after surgery (*P* < 0.001). There was also a significant interaction between time and the treatment group (*P* < 0.01). The total symptom score of groups A and B (5.85 ± 3.97 and 5.99 ± 4.67, respectively) was significantly lower than that of group C at 2 hours postsurgery (7.35 ± 4.86, *P* < 0.05), especially in tearing and pain (*P* < 0.05). The level of corneal oedema at 24 hours postsurgery was also statistically significantly different between the three groups (*P* < 0.001), and the post hoc test showed that groups A and B were lower than group C (*P* < 0.01).

**Conclusion:**

Silicon hydrogel BCLs applied immediately after SMILE surgery can relieve postsurgical symptoms of tearing and pain, improving overall patient comfort, and reduce corneal oedema. This trial is registered with ChiCTR-ONRC-13003114. *Precis*. The application of silicone hydrogel bandage contact lenses immediately after SMILE surgery has the potential to improve patient comfort, corneal healing, and patient satisfaction following SMILE.

## 1. Introduction

Refractive surgery has seen continual improvement over the last few decades, with a number of new procedures now being performed worldwide. Advances in technology have made refractive surgeries less invasive, more predictable, and capable of achieving better visual outcomes and less patient discomfort. One of the latest surgical techniques, small incision lenticule extraction (SMILE), has become popular due to its excellent predictability, stability, small incision required, and reduced complications [1–3]. However, despite its advantages, quite a few number of patients complain of ocular discomfort after surgery, including tearing, difficulty with eye opening, and so on. Improved methods for reducing the stimulative symptoms associated with SMILE require continuous exploration.

Bandage contact lenses (BCLs) are often used to promote corneal wound healing and reduce patient discomfort after laser refractive surgery [4–6], particularly following procedures such as photorefractive keratectomy (PRK) and laser epithelial keratomileusis (LASEK). As there are many different types of contact lens materials and designs available on the market, researchers have been investigating the most suitable type of contact lens to be used after laser refractive surgery [7–9]. Conventional hydrogel BCLs had low oxygen transmissibility, which might potentially cause hypoxic complications of the cornea when prescribed using a continuous wear modality [6, 10]. Silicone hydrogel (SiH) materials, on the other hand, have a higher oxygen transmissibility property, which has been shown to enhance corneal wound healing after refractive surgeries [11–13], and have been approved by the Food and Drug Administration (FDA) to be used as a BCL for a continuous wear modality [14].

We identified no literature reporting on the clinical application of SiH BCLs after SMILE surgery so far. Hence, the purpose of this research was to investigate the safety and efficacy of immediate use of SiH BCLs after SMILE surgery in reducing patients' discomfort, such that to provide the clinical basis for its use in the postoperative setting.

## 2. Method and Subjects

### 2.1. Participants

This prospective randomized controlled study was approved by the ethics committee of the Eye and ENT Hospital affiliated with Fudan institutional review board and was carried out in accordance with the tenets of the Declaration of Helsinki. After a detailed explanation of the study design, written informed consent was obtained from all participants.

The sample size for this study was determined using the formula: $n=\left(\Psi^{2}\left(\sum S_{i}^{2}/K\right)\right)/\left[\sum {\left(X_{i}-\;\bar{X}\right)}^{2}/\left(K-1\right)\right]$ based on the statistics provided in the referenced article [10], where the mean symptom score was *X*_*i*_ = 0.76, 0.31, and 0.20, and the reference standard deviation was *S*_*i*_ = 1.19, 0.55, and 0.52. Assuming an alpha of 0.05 and a beta of 10%, the sample size was calculated as *n* = 2.57^2^(1.989/3)/[0.176/(3 − 1)] = 49, assuming a lost to follow-up rate of 20%, and 60 participants are required in each group, making a total of 180 participants.

One hundred and eighty patients who presented to the Eye and ENT Hospital of Fudan University for SMILE surgery between December 2017 and September 2018 were recruited in the study. The inclusion criteria for this study were as follows: aged 18 years and above and cessation of contact lens wear for at least 2 weeks for soft contact lens wearers, 8 weeks for rigid contact lens wearers, and over 3 months for orthokeratology contact lens wearers. Subjects were excluded if their tear film break up time is less than 10 seconds or if they presented with any anterior ocular diseases, such as keratoconus and inflammatory eye diseases, systemic connective tissue disease or autoinflammatory diseases, and unstable mental health.

The participants were randomly allocated to three groups. There were 178 subjects (354 eyes) included in the analysis: group A had 59 subjects (118 eyes), group B had 60 subjects (120 eyes), and group C had 59 subjects (116 eyes) who served as the control. Two subjects were excluded as they failed to show up at follow-up visits.

The average age of all the subjects was 27.1 ± 5.9 years (range, 18 to 40 years), with 113 being male and 65 being female. The average refractive error of all participants was −5.18 ± 1.72 DS (range, −9.50 to −0.50 DS). The average presurgical astigmatism of all the participants was −0.92 ± 0.67 DC (range, −6.00 to −0.25 DC). The average corneal ablation depth (thickness of lenticule) was 114.85 ± 24.81 *μ*m (range, 50 to 160 *μ*m). There were no significant differences in the demographic data between the three groups, as represented in Table 1.

### 2.2. Study Protocol

All participants underwent routine preoperative examinations including anterior eye examination, refraction, intraocular pressure, and corneal topography. The SMILE procedure was performed as described by Zhao et al. [15, 16], by an experienced surgeon who performs over 2000 SMILE surgeries annually. All participants were given 3 drops of topical anaesthesia (0.4% bupivacaine hydrochloride) prior to laser surgery. SMILE was performed using the 500 Hz VisuMax® laser system (Carl Zeiss Meditic AG). The lenticule was removed via a 2 mm arcuate incision at the superior limbus. The corneal cap thickness was set as 120 *μ*m, and the lenticule diameter was between 6.0 and 6.8 mm, adjusted to the thickness of the lenticule.

Following surgery, all participants were given 1 drop of Tobradex (Alcon, USA), and participants in groups A and B were then fitted with a SiH BCL (Acuvue Oasys, senofilcon A, 38% water content, 147 Dk/L, 14.0 mm diameter, 8.80 mm base curve, and 0.07 mm central thickness, Johnson and Johnson, USA). Lens fitting was evaluated immediately after the insertion of BCLs, with a good centration and modest movement being confirmed in all cases.

Group A participants had their contact lenses removed 8 hours after surgery, and group B participants had their contact lenses removed 24 hours after surgery with sterilized microforceps. All participants were given topical tobramycin and fluorometholone acetate eye drops (0.1%) every 3 hours after surgery on the operation day. Lens stability and movement was confirmed 8 hours after surgery, and all the subjects were reviewed at 24 hours after operation. Uncorrected visual acuity (UCVA) was measured using a Snellen visual chart and recorded as logMAR.

### 2.3. Scoring

Participants' subjective symptoms and postoperative comfort were measured using a self-administered standardized survey [6] conducted at 2 hours, 4 hours, 8 hours, and 24 hours after surgery. Participants were required to return the questionnaire at the 24 hour review time. The questionnaire inquired about 8 symptoms, including photophobia, tearing, pain, foreign body sensation, burning, blurred vision, sting, and dry eyes. Each symptom was scored using a grading scale of 0–3 points, with 0 being no symptoms, and 1, 2, 3 indicating mild, moderate, and severe symptoms, respectively. The total score was recorded as the final symptom score.

Participants' eyes were examined for corneal oedema and conjunctival redness at the 24-hour follow-up visit. The severity of these signs was assessed under a slit-lamp microscope by the same ophthalmologist who was masked from the participant's group allocation. The clinical signs were scored using a 0–3 point scale. The higher the score, the more severe the corneal oedema and conjunctival injection.

### 2.4. Statistical Analysis

Statistical analyses were conducted using SPSS 20.0. Data are expressed as mean ± SD, frequency, and percentage. Participants' baseline demographic details were compared using one way-ANOVA and chi-square testing. Repeated measures ANOVA with an intragroup factor of time and an intergroup factor of treatment modality was applied to analyze the scores of patients' subjective symptoms and objective signs after surgery. Pairwise comparisons were carried out using Bonferroni corrections. *P* < 0.05 was considered statistically significant.

## 3. Results

### 3.1. Participant Subjective Symptom Score

**Figure 1:**
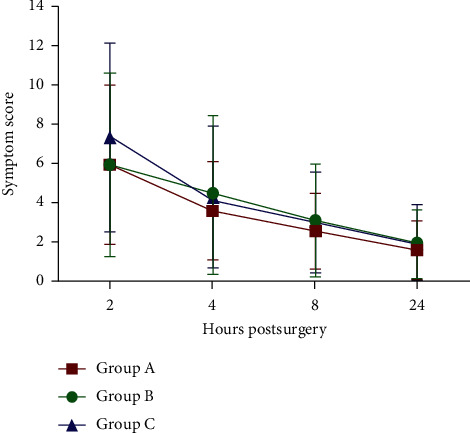
The total symptom scores after SMILE among the 3 groups at different postsurgery time points.

The overall subjective symptom scores are displayed in Table 2 and Figure 1. Repeated measures ANOVA showed a statistically significant time effect, which implicated a decline in symptoms over time after surgery (*P* < 0.001); the interaction between time and the treatment group was also statistically significant (*P* < 0.01). All three groups showed the highest symptom scores at 2 hours postsurgery, which declined at 4, 8, and 24 hours; scores in group C decreased faster than those in the other two groups from 2 to 4 hours postsurgery. Multiple comparisons showed that at 2 hours postsurgery, groups A and B had statistically significantly lower symptom scores than group C (*P*=0.012 and *P*=0.020, respectively), while no significant differences were seen between groups A and B (*P*=0.814). There were no significant differences in symptom scores between groups at all other time points (all *P* > 0.05).

The most reported postsurgical symptoms were blurred vision, photophobia, and tearing. At 2 hours postsurgery, among the 58 participants in group C, 68.8% experienced glare, 61.7% experienced tearing, 51.3% experienced varying degrees of pain, and 92.2% complained of blurred vision; both groups A and B had lower tearing (*P* = 0.027 and *P* = 0.005, respectively) and pain symptom scores (*P* = 0.036 and *P* = 0.048, respectively) than group C at that time.  Table 3 presents the symptom scores in all groups.

### 3.2. Corneal Oedema

On physical signs, there were also differences observed between the three groups. There was a statistically significant difference in corneal oedema at 24 hours postsurgery in all 3 groups (*P* < 0.001), with the post hoc test showing less corneal oedema in groups A and B compared to group C (*P* < 0.001 and *P*=0.001); there was no significant difference between groups A and B (*P*=0.472). Corneal oedema was recorded in 14.1% of the participants in group C, 4.9% in group B, and absent in group A. There were no statistically significant differences in limbal hyperemia between the three groups (*F* = 2.442, *P*=0.089).  Table 4 represents the scores of clinical signs in all three groups.

### 3.3. Uncorrected Visual Acuity Postsurgery

There were no statistically significant differences in UCVA between the three groups (*F* = 0.087, *P*=0.917). UCVA was −0.00 ± 0.11 in group A (BCL removed at 8 hours postsurgery), −0.03 ± 0.11 in group B (BCL removed at 24 hours postsurgery), and −0.00 ± 0.11 in group C (no BCL wear postsurgery).

## 4. Discussion

Corneal wound healing and postsurgical comfort are important issues concerning corneal refractive surgeries. The use of BCLs in refractive surgeries such as PRK and LASEK has been proven to be effective and safe [4, 5]. Studies have also shown that BCLs applied immediately after LASIK can reduce flap complications and patient discomfort after surgery [6, 17]. As SMILE is a relatively new type of refractive surgery, the clinical use of BCLs for the improvement of patients' comfort is not fully understood and requires further assessment.

The results of this study showed that the main subjective symptoms after SMILE surgery were blurred vision, photophobia, tearing, and foreign body sensation. The symptom scores were highest at 2 hours postsurgery, which then gradually reduced over time. This reduction in symptom scores over time reflects what is already known and expected and is similar to the findings reported by previous studies investigating other types of corneal refractive surgery. However, in the present study, the observed trend was slightly different to previous studies. For example, Orucov et al. [6] reported that pain, tearing, and discomfort are most prominent at 4 hours after LASIK when compared to 1 hour postsurgery. O'Doherty et al. [18] also found that Epi-LASIK and PRK patients who wore BCLs postsurgery experienced a gradual rise in pain symptoms in the early postoperative period, which peaked at 4 hours postsurgery, whilst their LASIK patients experienced the greatest discomfort at 2 hours postsurgery. This postsurgical pain is often related to corneal nerve ending exposure as well as the mechanical friction of the eyelid over the surgical site; however, this pain often subsides at 24 hours postsurgery. This difference in symptom change over time could be due to that SMILE, and unlike most refractive surgeries, it is a flapless laser refractive procedure, with the benefit of creating only a small corneal wound with minimal corneal nerve damage. However, the separation of the anterior and posterior stroma, as well as the process of removing the lenticule has the potential to cause disruption to the surrounding corneal tissue, thereby affecting the corneal surface [19] and inducing patients' postsurgical discomfort.

In the present study, patients who wore BCL after surgery had lower scores of total symptoms and tearing and pain at 2 hours than those without BCLs. This indicated that the utilization of a SiH BCL can help alleviate early stage pain and tearing symptoms from the SMILE procedure. Orucov et al. [6] showed that BCL application after LASIK was effective in reducing early burning, pain, and tearing symptoms; patients who wore BCLs for 1 hour or 1 day postsurgery had significantly lower symptoms than those without BCLs at 4 hours and the following morning. Ahmed and Breslin [20] showed that most LASIK patients who wore BCLs within 3 hours postsurgery reported better ocular comfort than those without BCLs. The effect of using BCLs to relieve pain and discomfort after LASEK surgery was previously confirmed [13, 21]. Xie et al. [13] found that LASIK patients who wore silicone hydrogel contact lenses postsurgery had reduced pain and photophobia than hydrogel contact lenses. BCLs can aid in the protection of the exposed corneal wound and nerve endings, reducing the irritation that arises from blinking. The present study demonstrated that the wearing of SiH BCLs for a short period after SMILE can reduce ocular symptoms and improve overall comfort of patients, which shows the novelty of this study.

This study also found that the BCL wearing group had lower corneal oedema scores than the no BCL wearing group at 24 hours postsurgery. SMILE has been shown to yield low incidences of postoperative complications. Zhang et al. [3] reported no incidences of corneal swelling in their 45 subjects who had undergone SMILE surgeries. The present study found mild corneal oedema 24 hours postsurgery in some patients, which may be partly caused by the suction ring used during surgery. The BCLs used in the present study utilized a silicone hydrogel material, which has a high oxygen transmissibility. This effectively avoided hypoxic complications such as limbal injection and corneal swelling from overnight lens wear as demonstrated by Fonn et al. [10, 22, 23] who found reduced levels of corneal oedema with SiH CLs (range, 2%–5%) when in contrast with conventional low Dk lens (10% to 15%). It has been shown that in LASIK patients, the speed of corneal epithelium healing was faster in SiH CL wearers (4.1 ± 0.3 days) when compared to hydrogel lens wearers (4.3 ± 0.8 days) [13]; whilst another study showed that eighty percent of eyes with a SiH CLs versus 63% of eyes with hydrogels had grade 1 epithelial recovery at 5 days postsurgery [12]. Gao et al. [17] also found that the corneal thickness of eyes in silicone hydrogel lens were thinner than those wearing hydrogel lens at 1 day after Sub-Bowman keratomileusis (SBK) surgery. More investigations are needed to confirm if SiH BCLs may further assist, as the results of this study suggested, in preventing or alleviating corneal oedema related with surgery procedures after SMILE.

The present study used a SiH lens with a base curve (BC) of 8.80 mm (Acuvue Oasys, Johnson and Johnson). The lens demonstrated excellent centration with adequate movement, which was suitable for post-SMILE patients. Previous studies have examined the applications of the same lens for post PRK and LASEK conditions and have compared it with two other SiH CLs; the results showed that this lens was superior when it comes to patients' comfort [24]. A comparison between the two base curves available for this lens has also been carried out: the 8.80 mm BC was found to be more suitable for the postsurgical flatter cornea as compared to 8.40 mm BC and was effective in alleviating postsurgical pain as well as reducing the incidence of lens loss [4]. Plaka et al. [11] found that the 8.80 mm BC lens was also superior to the 8.40 mm BC lens in terms of UCVA at 4 days after operation in their Epi-LASIK patients.

To our knowledge, this is the first randomized clinical trial aimed at investigating the efficacy of SiH BCLs used postoperatively to reduce patient discomfort in the population underwent SMILE surgery, which provide insightful findings for clinical practice in this field. Second, the outcome assessor was blinded to the group allocation, therefore, the credibility of results was ensured. Moreover, the effectiveness of SiH BCLs was comprehensively evaluated, which has been rarely reported in previous study.

The limitation in the current study is that the subjective symptoms were only observed in a short time frame of 2–24 hours postsurgery.

To conclude, short-term wearing of SiH BCLs after SMILE is effective in relieving pain and tearing symptoms and thus improves patient ocular comfort. It is recommended to use the bandage contact lens for short-term, 4–24 hours, after SMILE, which could improve patient satisfaction. In addition, bandage contact lens wearing may also reduce corneal oedema after surgery, which could be further investigated.

## Figures and Tables

**Table 1:** Participants' baseline demographics.

Group	*n*	Age	Gender	Spherical power (D)	Cylindrical power (D)	Ablation depth (*μ*m)
			Females (%)			
A	59	27.17 ± 5.91	38 (62.3)	5.15 ± 1.77	0.94 ± 0.58	114.98 ± 23.27
B	60	26.98 ± 5.91	36 (60.0)	4.94 ± 1.54	0.87 ± 0.67	112.55 ± 25.48
C	59	27.23 ± 6.05	39 (66.1)	5.45 ± 1.80	0.96 ± 0.77	116.88 ± 25.74
*F*/*χ*^2^		0.026	0.545	2.419	0.474	0.767
*P*		0.974	0.761	0.091	0.623	0.465

(Mean ± SD, *N* = 178). Group A: wore BCLs for 8 hours, group B: wore BCLs for 24 hours, and group C: no BCL wear.

**Table 2:** Comparison of the total symptom scores after SMILE between the 3 groups at different postsurgery time points.

Group	*n*	2 h	4 h	8 h	24 h	Time effect	Group effect	Time *x* group
						*F*	*F*	*F*
A	59	5.85 ± 3.86	3.63 ± 2.52	2.59 ± 1.71	1.62 ± 1.37			
B	60	5.99 ± 4.35	4.43 ± 3.62	3.11 ± 2.55	1.98 ± 1.49			
C	59	7.35 ± 4.44^*∗*^	4.30 ± 3.31	3.02 ± 2.36	2.03 ± 1.60	310.182	3.055	3.877
*F*		4.534	2.177	1.807	2.618			
*P*		0.011^*∗*^	0.115	0.166	0.074	0.000^*∗∗*^	0.048	0.001^*∗*^

(Mean ± SD, *N* = 178). Group A: wore BCLs for 8 hours, group B: wore BCLs for 24 hours, and group C: no BCL wear. ^*∗*^*P* < 0.05;^*∗∗*^*P* < 0.001.

**Table 3:** The scores of symptoms after SMILE in all 3 groups.

Symptom	Time after surgery (h)	Group A	Group B	Group C	*F*	*P*
Blurred vision	2	1.46 ± 0.74	1.52 ± 0.75	1.53 ± 0.80	0.306	0.736
	4	1.17 ± 0.60	1.29 ± 0.69	1.19 ± 0.70	1.079	0.341
	8	0.99 ± 0.61	1.15 ± 0.64	1.00 ± 0.57	2.534	0.081
	24	0.80 ± 0.59	0.92 ± 0.60	0.82 ± 0.52	1.574	0.209

Photophobia	2	1.18 ± 1.02	1.03 ± 1.13	1.31 ± 1.14	1.755	0.174
	4	0.69 ± 0.87	0.59 ± 0.77	0.67 ± 0.77	0.434	0.649
	8	0.34 ± 0.57	0.34 ± 0.60	0.37 ± 0.52	0.106	0.900
	24	0.16 ± 0.36	0.11 ± 0.31	0.24 ± 0.43	3.686	0.018^*∗*^

Tearing	2	0.92 ± 1.04	0.83 ± 1.11	1.23 ± 1.16	4.474	0.012^*∗*^
	4	0.38 ± 0.63	0.47 ± 0.79	0.36 ± 0.64	0.755	0.471
	8	0.07 ± 0.25	0.15 ± 0.40	0.10 ± 0.32	1.754	0.175
	24	0.04 ± 0.20	0.06 ± 0.23	0.05 ± 0.22	0.134	0.875

Pain	2	0.50 ± 0.73	0.61 ± 0.81	0.83 ± 0.96	3.524	0.031^*∗*^
	4	0.18 ± 0.43	0.36 ± 0.74	0.31 ± 0.55	2.791	0.063
	8	0.18 ± 0.42	0.19 ± 0.47	0.29 ± 0.56	1.844	0.160
	24	0.04 ± 0.20	0.06 ± 0.23	0.08 ± 0.27	0.727	0.484

Foreign body sensation	2	0.44 ± 0.72	0.64 ± 0.84	0.70 ± 0.93	2.977	0.049^*∗*^
	4	0.38 ± 0.53	0.61 ± 0.75	0.53 ± 0.67	3.683	0.027^*∗*^
	8	0.30 ± 0.53	0.53 ± 0.63	0.42 ± 0.62	4.158	0.016^*∗*^
	24	0.20 ± 0.44	0.40 ± 0.58	0.25 ± 0.45	4.769	0.009^*∗*^

Burning	2	0.42 ± 0.70	0.44 ± 0.81	0.59 ± 0.76	1.529	0.218
	4	0.07 ± 0.25	0.25 ± 0.61	0.31 ± 0.58	6.173	0.003^*∗*^
	8	0.06 ± 0.24	0.14 ± 0.47	0.14 ± 0.34	1.560	0.212
	24	0.01 ± 0.09	0.05 ± 0.22	0.08 ± 0.27	3.378	0.037^*∗*^

Sting	2	0.39 ± 0.73	0.46 ± 0.85	0.56 ± 0.83	1.165	0.313
	4	0.15 ± 0.40	0.30 ± 0.69	0.32 ± 0.60	2.968	0.053
	8	0.16 ± 0.39	0.19 ± 0.47	0.21 ± 0.46	0.349	0.706
	24	0.04 ± 0.27	0.05 ± 0.22	0.08 ± 0.27	0.657	0.519

Dry eyes	2	0.43 ± 0.60	0.49 ± 0.67	0.59 ± 0.82	1.567	0.210
	4	0.47 ± 0.68	0.51 ± 0.62	0.67 ± 0.65	3.039	0.049^*∗*^
	8	0.46 ± 0.53	0.46 ± 0.70	0.52 ± 0.63	0.389	0.678
	24	0.35 ± 0.51	0.39 ± 0.55	0.50 ± 0.63	1.919	0.148

(Mean ± SD, *N* = 178). Group A: wore BCLs for 8 hours, group B: wore BCLs for 24 hours, and group C: no BCL wear. ^*∗*^*P* < 0.05.

**Table 4:** Clinical sign scores at 24 hours after surgery in the 3 groups.

Groups	*n*	Limbal hyperemia (% of subjects)	Corneal oedema (% of subjects)
A	59	0.03 ± 0.16 (2.5)	0.00 ± 0.00 (0.0)
B	61	0.12 ± 0.32 (11.0)	0.03 ± 0.18 (4.9)
C	58	0.07 ± 0.24 (5.6)	0.22 ± 0.42 (14.1)
*F*		2.442	10.780
*P*		0.089	0.000^*∗∗*^

(Mean ± SD, *N* = 178). Group A: wore BCLs for 8 hours, group B: wore BCLs for 24 hours, group C: no BCL wear. ^*∗∗*^*P* < 0.001.

## Data Availability

The data used to support this study are available from the corresponding author upon request.
